# Assessment of the Inner Surface Microstructure of Decellularized Cortical Bone by a Scanning Electron Microscope

**DOI:** 10.3390/bioengineering6030086

**Published:** 2019-09-19

**Authors:** Heonuk Jeong, Jungo Asai, Takashi Ushida, Katsuko S. Furukawa

**Affiliations:** 1 Department of Bioengineering, University of Tokyo, Hongo 7-3-1, Tokyo 113-8654, Japan; jeong@biomed.t.u-tokyo.ac.jp; 2 Department of Mechanical Engineering, University of Tokyo, Hongo 7-3-1, Tokyo 113-8654, Japan; asai@biomed.t.u-tokyo.ac.jp (J.A.); ushida@mech.t.u-tokyo.ac.jp (T.U.)

**Keywords:** decellularized bone, surface topography, bone microstructure, endocortical surface, scanning electron microscope

## Abstract

The microstructural changes of bones, which form a hierarchy of skeletal tissue, vary, depending on their condition, and are affected by the behaviors of bone cells. The purpose of this study is to assess the microstructural changes in the inner femoral surface of Sprague Dawley rats according to the conditions using a scanning electron microscope. Microstructural differences on the endocortical surface were observed in the characteristics of osteocytic canaliculi, bone fibers, and surface roughness, showing a rougher surface in old adults and an osteoporosis model by quantitative comparison. These results could be helpful for developing a basic understanding of the microstructural changes that occur on the bone surface under various conditions.

## 1. Introduction

In the human body, bone is designed to protect organs in the body, store bone marrow, and provide support for movement of the body. Cortical bone constitutes the outer region of bone and has a hierarchical structure, with components such as minerals and collagen. At the microstructure level, the cortical bone, composed of compacted osteon units, exhibits dense aggregations of the lamella of several micrometers in thickness, consisting of mineralized collagen fibrils, providing the micro- and nanostructure [1,2]. The hierarchical structure of the bone is maintained through matrix resorption and formation by cells, including osteoclasts, osteoblasts, and osteocytes [3,4,5]. 

Osteocytes embedded in the bone matrix spread their canaliculi to communicate with other cells, i.e., the canaliculi of osteocytes extend to the interfaces of the cell and bone matrix. On the endocortical surface, osteoclasts and osteoblasts are recruited from the bone marrow or blood vessels to play their roles in bone remodeling in degradation and mineralization of the bone matrix [6,7]. Should the actions of the cells in bone remodeling become unbalanced, excessive bone resorption by osteoclasts will lead to a decreased bone mineral density and result in osteoporosis [8]. In this case, the resorption cavity will gradually increase, resulting in a loss of bone mass and toughness, while inducing structural changes in the bone matrix at the microscale.

However, it is still unclear how much structural change occurs on the interface of the cell and bone matrix in vivo. In previous studies on the bone structure on a micro- and nanoscale, Pazzaglia et al. observed the structure of the femoral bone from rabbits (New Zealand White rabbit, 8 months), using scanning electron microscopy (SEM) observation to examine the density and the size of canals and collagen fiber bundles on the endocortical surface [9]. Other studies have considered the vascular canals in age-related changes of the rabbit femur. However, features of the cell-bone matrix interface have not been evaluated [10]. In addition, the osteocytic canalicular thickness of humeri from 15-week-old mice was measured using an electron microscope [11]. Moreover, at the nanoscale, three-dimensional hierarchical structures of minerals and collagen in human cortical bone were also analyzed [12]. However, those studies did not consider the interface features or the diversity of bone conditions.

We hypothesized that there will be microstructural changes in the matrix–cell interface of the bone if it displays osteoporosis or is age-specific because of the difference in the balance of cells in bone remodeling. In this study, we aim to characterize the endocortical surface of the cell–matrix interface in various conditions, including age-specific and osteoporosis conditions. We prepared four types of femoral bones from Sprague Dawley (SD) rats of age-specific and osteoporosis models. Through decellularization of the samples, we could easily observe the structure of the bone matrix and evaluate the structural features and the roughness of the endocortical surface using SEM. By analyzing the structural characteristics of the surface, we were able to identify the microstructural differences according to the model. These results can help us understand the structural changes of the bone and act as a basic study for bone regeneration using structural applications. Furthermore, it is expected that the study will be applied to bone disease diagnostics by simple observation of the bone.

## 2. Materials and Methods 

### 2.1. Femoral Bone Harvest

Female SD rats were purchased from CLEA Japan, Inc. (Tokyo, Japan), and were prepared in four groups as 2–4 weeks old for weanlings (WL), 8–10 weeks old for young adults (YA), 8–10 weeks old with ovariectomy for the osteoporosis model (OVX), and over 40 weeks old for old adults (OA). The OVX model was the rat past three weeks after ovariectomy. In each group, three rats were used to harvest femoral bones. The use of animals in this study was in compliance with the ethics set up by the University of Tokyo. Rats were euthanized using carbon dioxide gas, as described by protocols set by the University of Tokyo for femoral bone isolation. The femurs were isolated immediately after euthanization; surrounding flesh and muscle were also removed. The bones were washed with phosphate-buffered saline (PBS) and transferred for the next step of the experiment.

### 2.2. MicroCT Observation 

Harvested femoral bones from four different models of SD rats were fixed in 10% formalin for 2 days and transferred to 70% ethanol. Then, they were scanned on 3D micro X-ray CT (R_mCT2, Rigaku, Tokyo, Japan). Before observation of the samples, a calibration scan using a hydroxyapatite phantom rod in the water-filled tube was performed. All scans were conducted using the same condition at a 90 kV voltage, 160 µA current, and 26 s integration time. 3D scanning images were then reconstructed. The microCT images were obtained with the field of view of φ 5 × Height 5 mm and the resolution of 512 × 512 pixels.

### 2.3. Decellularization Treatment

Decellularized bones were used to observe the endocortical surface of the femoral bone in each model. SD rats were sacrificed on design time and femoral bones were harvested. The bones were washed with PBS and then embedded in 0.5% (w/v) SDS and 0.1% (w/v) NH_4_OH in PBS for the removal of cells. After decellularization, the bones were washed with distilled water and fixed in 10% formalin. 

### 2.4. Hematoxylin and Eosin Staining

Hematoxylin and eosin (H&E) staining was performed on the sections of the bone samples to confirm the removal of cells and the remaining bone matrix after decellularization treatment. Decellularized samples were embedded in 10% ethylenediaminetetraacetic acid (EDTA) in PBS adjusted to pH 7.0 for one week at 4 °C, with slight agitation, in order to decalcify them. After washing them in distilled water, the samples were transferred to 30% sucrose solution and incubated at 4 °C for 3 days, changing the solution every 24 hours. Samples were then embedded in an optimum cutting temperature (OCT) compound (Sakura Finetek, Tokyo, Japan). The samples were cryosectioned at an 8 µm thickness and stained with H&E solution. Representative stained sections were observed using an optical microscope.

### 2.5. Scanning Electron Microscopy Observation

To observe the microstructure of the inner surface, decellularized femoral bones were cut and both tips of the epiphysis were removed to leave the midshaft part. Then, the remaining parts were cut and fixed with carbon tape on the sample stage so that the inner surface of the shaft could be observed. Afterwards, the specimens were prepared with an osmium coating. SEM observation was carried out using a high-resolution SEM (JSM-7000F, JEOL).

### 2.6. Image and Statistical Analysis

To measure the topographic features on the surface (density, size, distance between two closest holes, and qualitative roughness score), data from SEM images were analyzed by ImageJ. Five SEM images of each sample were randomly chosen for the measurement. Statistical significance was determined by the two-sided Student’s *t*-test and *p*-values less than 0.05 were considered significant. 

## 3. Results and Discussion

**Figure 1:**
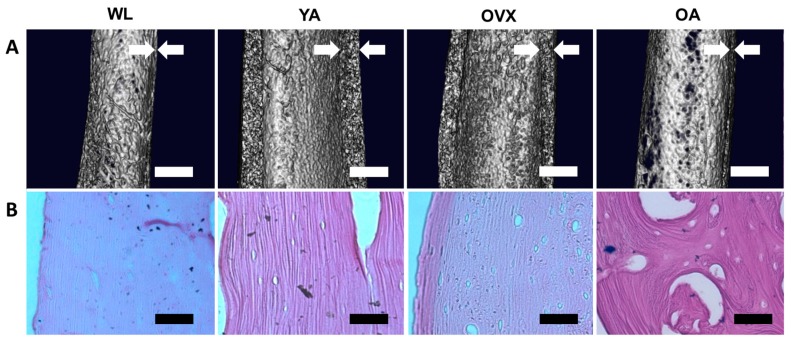
Macro-observation of the femoral bone shaft. (**A**) MicroCT images of the midshaft part of the femoral bones in four different models. White arrows indicate the cortical thickness. Scale bar: 1 mm. (**B**) Hematoxylin and eosin (H&E) staining images of the decellularized cortical bones. The extracellular matrix is stained in violet, suggesting that the bone matrix remains after decellularization treatment, while cells are removed. Scale bar: 100 µm.

We prepared four models of SD rats: 2–4 weeks old for weanlings (WL), 8–10 weeks old for young adults (YA), 8–10 weeks old with ovariectomy for the osteoporosis model (OVX), and over 40 weeks old for old adults (OA). Ovariectomies were conducted on OVX rats to increase the bone resorption increase, resulting in bone loss [13]. Prior to the observation, femoral bones isolated from each model were decellularized using sodium dodecyl sulfate (SDS) and ammonium hydroxide (NH_4_OH) solution for easy observation of the surface of samples. This decellularization method is effective for removing cell residues from hard tissue, such as bone, whilst conserving the original structural features [14]. After decellularization treatment, the femoral shaft in each model was first observed by microCT. 3D images were reconstructed by cutting the femoral shaft in half longitudinally to confirm the thickness of the femoral cortical bone (Figure 1A). Cortical bones in YA and OVX were thicker than OA and WL, with YA being the thickest. OVX and OA were thinner than YA, suggesting that cortical thickness is affected by osteoporosis and age. Cortical bone was smallest in WL due incomplete bone development. Likewise, studies on the measurement of periosteal perimeter thickness and cortical thickness according to age and sex have shown that as the age increases, the cortical thickness decreases and the periosteal perimeter increases [15,16].

Next, decellularized femur samples were sectioned longitudinally and stained with a hematoxylin and eosin (H&E) solution. Due to the decellularization treatment, cells were not observed in either the cortical bone or the bone matrix; however, the bone matrix was still observable (Figure 1B). Moreover, the larger size of cavities was observed more clearly at OA. The absence of cells in the matrix allowed easy observation of the structural changes on the endocortical surface.

**Figure 2:**
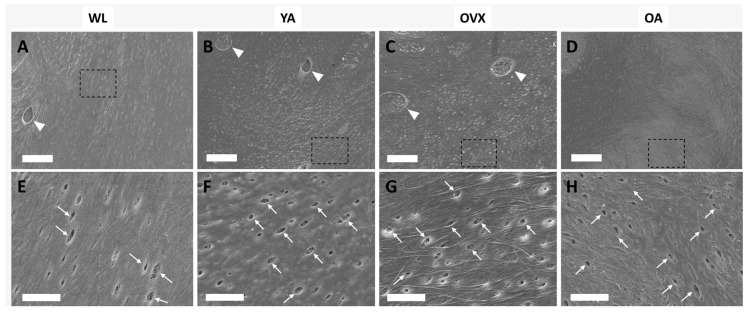
Scanning electron microscopy (SEM) images of the endocortical surface of the femoral bone in the four models (**A**,**E**, weanlings (WL); **B**,**F**, (young adults) YA; **C**,**G**, osteoporosis model (OVX); **D**,**H**, (old adults) OA). The bottom images are enlargements of the dashed boxes in the upper images, respectively. Triangles indicate the traces of osteocytes, and arrows indicate the canaliculi holes. (**A**–**D**) Scale bar: 20 µm. (**E**–**H**) Scale bar: 5 µm.

To assess the topographical differences of bone matrix interfaces in the four models, we observed the endocortical surface of the femoral shaft by SEM. Many small holes were observed on the surface of all models (Figure 2). In addition, traces (triangles) sporadically existed in the shape of an ellipse, and small holes (arrows) were also observed. The small holes, when viewed in terms of size and density, could be osteocyte canaliculi holes, and the traces could be where osteocytes were present [9,17]. The canaliculi of the osteocyte detect mechanical loading and communicate with other cells on the bone surface [18]. Therefore, the number of canaliculi varies, depending on the condition of the bone, which can change the surface topography. 

Since each hole is approximately less than 1 µm^2^ in size and these canaliculi holes could be one of the important factors determining the topographic features of the surface, we measured the various elements of the holes in each model (density, size, and distance between two closest holes).

**Figure 3:**
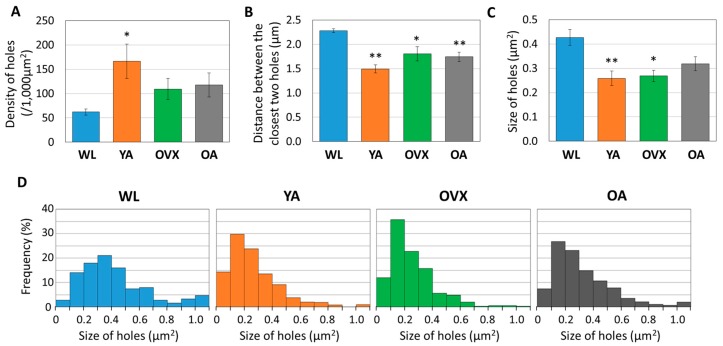
Histograms measuring (**A**) density, (**B**) distance between the closest two holes, and (**C**) size of 361 to 696 holes from three different individuals in each group. Values indicate the means ± standard error (S.E.). Significant differences between the WL group are indicated by *, *p* < 0.05 and **, *p* < 0.01. (**D**) Graph showing the distribution of hole sizes as proportions in four different models. In total, 361 to 696 of the holes are plotted according to their size.

We found that there were some differences between models. First, the density of the holes was lowest in WL and highest in YA (Figure 3A). Moreover, there was a significant difference between the two groups, with OVX and OA lying between these two groups. The distance observed between holes was opposite to that of density (Figure 3B). These results suggest that the number of canaliculi holes is low in WL because the bone is in the process of growing and it decreases when osteoporosis occurs or when reaching an old age. 

When comparing the sizes of the holes, the largest value was recorded in the WL group, showing a significant difference from the other models (Figure 3C). However, there was no significant difference in the other models. When the distribution according to the size of the hole is compared, it can be seen that the ratio of the large hole (>0.5 µm^2^) in WL is also higher than in the other groups (Figure 3D). These results suggest that morphological changes of canaliculi during bone growth may occur.

**Figure 4:**
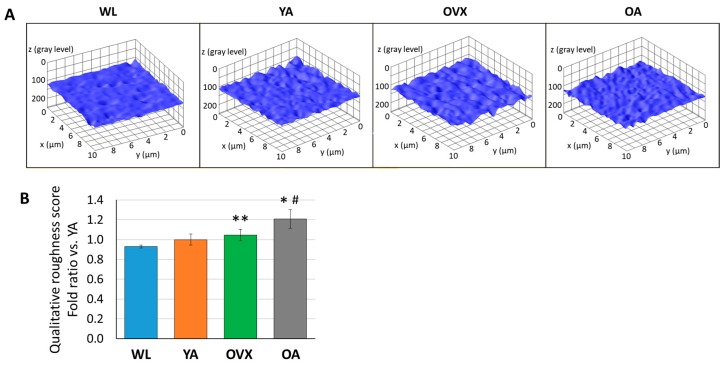
(**A**) Representative 3D plot images of surface roughness by ImageJ from SEM images of each model. (**B**) Histograms of qualitative roughness score from three different individuals in each group. Values are normalized by that of a young adult and indicate the means ± standard error (S.E.). Significant differences between the WL group are indicated by *, *p* < 0.05 and **, *p* < 0.01, and those between the YA group are indicated by ^#^, *p* < 0.05.

In order to compare the roughness of the endocortical surface, we extracted 3D structures from the SEM images of each group using the image analysis software (ImageJ, National Institutes of Health, USA) and also calculated the qualitative roughness score (QRS) with an ImageJ plugin (SurfCharJ, http://www.gcsca.net/IJ/SurfCharJ.html) for the quantitative comparison of each group. Since SEM images are obtained by detecting the secondary electrons from the sample surface as intensity, the spread of the distribution of gray values at each point of the SEM image is used as an index of the surface roughness for each group [19,20]. In 3D reconstructed images, the surface of WL seems smoother than that of other groups (Figure 4A). As a result of the roughness comparison, OVX and OA showed higher QRS than WL and YA (Figure 4B). Furthermore, the average of QRS was in the order of WL, YA, OVX, and OA. OA showed significantly higher values than WL and YA (*p* < 0.05), and there was also a significant difference between OVX and WL (*p* < 0.01). Studies related to osteoclastogenesis have shown that when RAW264.7 cells were cultured on the surfaces with different roughnesses, osteoclast differentiation increased on the rough surface more than on the smooth substrate in vitro [21,22,23]. On the other hand, a study on the osteogenic differentiation of human osteoblastic cells in polyetheretherketone implantable material demonstrated that cell proliferation and differentiation increased on the moderate surface implant compared with smoother and rougher groups [24]. Therefore, the results which showed relatively higher QRS on OVX and OA than WL and YA could imply that the cell balance in bone remodeling and the cell–bone matrix interface changes are related in vivo.

Although this study compared the topographical features of the endocortical surface of four models, micro- and nanostructural changes due to time elapsing in osteoporosis development or aging were not studied. Other studies have been conducted by groups such as Wronski et al., who reported bone loss by measuring data such as the calcification rate, trabecular bone volume, and bone formation rate in SD rats for 180 days post-ovariectomy [13]. Similar to this, future works include a quantitative analysis of endocortical surface characteristics as time passes in experimental models based on our present results. Data such as the orientation and thickness of bone fibers, osteocytic canalicular arrangement, and the rigidity of endocortical bone could help determine the physical characteristics of surfaces. The use of modern observation techniques, such as nanoCT and focused ion beam-SEM, will allow us to quantify them [12,25,26].

Bone diseases such as osteoporosis are difficult to diagnose prior to the onset of the disease. However, early diagnosis of the bone disease will become feasible if we can observe the microstructural changes in the bone before disease occurs. We expect that the results of endocortical surface changes in various models can be used as an indicator of early diagnosis.

Moreover, to promote osteoinductivity in vitro, studies on structural characteristics of bones, such as the pore shape, size, distribution, surface topography, and roughness on the micro and nanoscale, have been conducted because of the importance of the structural understanding of bones [27,28,29,30]. However, research on the surface properties of implanted scaffolds that imitate the bone microstructures in vivo has rarely been conducted. It is hoped that the results of this study can be applied to scaffolds to improve the interface between the cell and matrix to provide a microenvironment that mimics the real environment.

## 4. Conclusions

In conclusion, our study assessed the microstructural characteristics of the inner surface of the cortical bone from SD rats in four different models by SEM. Through observation of decellularied femoral bones, our results showed that the surface microtopography differs, depending on the bone model. The canaliculi holes of osteocytes were larger and there was a lower density in YA, whilst the cell surface interface was rougher in OA and OVX, as detected by the quantitative comparison. These changes on the surface might provide surface properties. In further studies, quantitative analysis of more indices of the bone on a micro- and nanoscale will further reveal the structural characteristics. Therefore, this work could be helpful for developing a basic understanding on the microstructure of and topographical changes that occur on the bone surface under various conditions. Furthermore, the outcome of the study may help as an index to detect changes on the bone surface in early bone diagnostics. In addition, if the surface microtopography of each bone model is mimicked and used as a topographical element for in vitro cell culture substrates, more meaningful applications will be possible for cell differentiation and behavior control in tissue regeneration.
